# Incretin responses to oral glucose and mixed meal tests and changes in fasting glucose levels during 7 years of follow-up: The Hoorn Meal Study

**DOI:** 10.1371/journal.pone.0191114

**Published:** 2018-01-11

**Authors:** A. D. M. Koopman, F. Rutters, S. P. Rauh, G. Nijpels, J. J. Holst, J. W. Beulens, M. Alssema, J. M. Dekker

**Affiliations:** 1 EMGO+ Institute for health and care research, VUmc, Amsterdam, the Netherlands; 2 Department of Epidemiology and Biostatistics, VUmc, Amsterdam, the Netherlands; 3 Department of General Practice & Elderly Care medicine, VUmc, Amsterdam, the Netherlands; 4 NNF Center for Basic Metabolic Research and Department of Biomedical Sciences, the Panum Institute, University of Copenhagen, Copenhagen, Denmark; 5 Julius Centre for Health Sciences and Primary Care, University Medical Centre, Utrecht, the Netherlands; 6 Unilever Research and Development, Vlaardingen, the Netherlands; Medical University Innsbruck, AUSTRIA

## Abstract

We conducted the first prospective observational study in which we examined the association between incretin responses to an oral glucose tolerance test (OGTT) and mixed meal test (MMT) at baseline and changes in fasting glucose levels 7 years later, in individuals who were non-diabetic at baseline. We used data from the Hoorn Meal Study; a population-based cohort study among 121 subjects, aged 61.0±6.7y. GIP and GLP-1 responses were determined at baseline and expressed as total and incremental area under the curve (tAUC and iAUC). The association between incretin response at baseline and changes in fasting glucose levels was assessed using linear regression. The average change in glucose over 7 years was 0.43 ± 0.5 mmol/l. For GIP, no significant associations were observed with changes in fasting glucose levels. In contrast, participants within the middle and highest tertile of GLP-1 iAUC responses to OGTT had significantly smaller increases (actually decreases) in fasting glucose levels; -0.28 (95% confidence interval: -0.54;-0.01) mmol/l and -0.39 (-0.67;-0.10) mmol/l, respectively, compared to those in the lowest tertile. The same trend was observed for tAUC GLP-1 following OGTT (highest tertile: -0.32 (0.61;-0.04) mmol/l as compared to the lowest tertile). No significant associations were observed for GLP-1 responses following MMT. In conclusion, within our non-diabetic population-based cohort, a low GLP-1 response to OGTT was associated with a steeper increase in fasting glucose levels during 7 years of follow-up. This suggests that a reduced GLP-1 response precedes glucose deterioration and may play a role in the etiology of type 2 diabetes mellitus.

## Introduction

Type 2 diabetes mellitus (T2DM) is characterized by hyperglycaemia, which results from a combination of insulin resistance, increased hepatic glucose production and pancreatic β-cell dysfunction[1, 2]. Recent evidence indicated that the gastrointestinal system, especially the incretin hormones, play a significant role in the pathophysiology of T2DM as well[3]. The growing interest in the use of incretin-based therapies and other interventions in early stages of the disease underscore the importance to determine the possible etiological role of the incretin hormones in T2DM.

During ingestion of food, the gastrointestinal incretin hormones, glucose-dependent insulinotropic polypeptide (GIP) and glucagon-like peptide-1 (GLP-1), are released from the gut into the bloodstream, where they bind to their specific receptors on the pancreas to regulate the amount of insulin and glucagon that is secreted to control blood glucose levels. The important role of the incretin hormones with regard to insulin secretion becomes apparent when the same amount of glucose is infused intravenously as opposed to orally ingested; oral glucose enhances insulin secretion much more than infused glucose: the so-called incretin effect[4, 5]. In healthy people, the incretin effect accounts for at least 50%[6] and up to 70%, of the total insulin secreted after oral glucose intake and therefore plays an important role in maintaining normal glucose regulation after meal intake[7, 8]. In T2DM patients, the incretin effect is severely reduced[9] and the impairment is thought to explain an important part of the impaired insulin secretion seen in T2DM[10].

Besides stimulating insulin secretion, through several pathways [11], the incretins have additional effects, both on postprandial metabolism, as well as on pathways that may exert long-term metabolic effects. In the postprandial state, GIP stimulates and GLP-1 suppresses glucagon secretion. In addition, GLP-1 lowers hepatic glucose production, increases glucose uptake in the muscles and slows gastric emptying[6, 12]. Long-term effects of the incretins include central nervous system effects to regulate appetite and food intake and also involve cardiovascular and neurotropic effects[6, 12]. Given the wide range of glucoregulatory actions of the incretin hormones and their importance in maintaining glucose homeostasis it was hypothesized that incretin responses are associated with changes in fasting glucose over time.

Up until now, only cross-sectional studies have investigated alterations in incretin responses, which have shown inconclusive results with GLP-1 and GIP being increased[10, 13, 14], decreased [15–22] or normal[16, 23–26] in T2DM and pre-diabetes. Meta-analyses of Calanna et al. 2013[27, 28] in T2DM patients, showed an overall preserved GIP[27] and unaltered GLP-1[28] secretion. However, there was substantial heterogeneity between studies, in particular for GLP-1 responses and stratified analyses showed different results for liquid versus solid meals. In a report on the cross-sectional data of our population-based cohort, GIP and GLP-1 responses (tAUC) were increased in T2DM patients, but not in persons with intermediate hyperglycaemia[29].

However, these cross-sectional findings should be interpreted carefully, because comparison between diseased and healthy individuals may be confounded by other metabolic alterations, such as differences in body mass index (BMI) and rate of gastric emptying in T2DM[30]. Prospective studies among non-diseased individuals could provide more insight into the role of incretins in the etiology of T2DM[31], by studying the relationship between incretin hormone response and glucose deterioration over time. Therefore, we conducted the first prospective observational study in which we examine the association between incretin responses to an oral glucose tolerance test (OGTT) and mixed meal test (MMT) at baseline and changes in fasting plasma glucose levels 7 years later, in individuals who were non-diabetic at baseline.

## Materials and methods

### Study population

The study participants were from the Hoorn Meal Study, which involves a population-based cohort of 40–65 year old men and women, all Caucasian, initiated in the year 2005[29, 32]. The study population consisted of 208 participants of which 194 persons were randomly invited from the general population in Hoorn, the Netherlands and 14 patients with T2DM were recruited from the regional Diabetes Care System. Details of the inclusion procedure were described earlier[29, 32]. After 7 years, a follow-up examination was carried out. Of the remaining 187 participants who were invited for the follow-up examination, 129 (69%) agreed to participate. For the current study, participants with T2DM at baseline (n = 5) or participants without glucose measures at follow-up (n = 3) were excluded, resulting in a study population of 121 subjects. All participants signed informed consent and the medical ethics committee of the VU University Medical Centre approved the study.

### Study procedure

At the baseline examination, participants received a 75g-OGTT and a standardized MMT after an overnight fast, on separate days, in random order, within 2 weeks. Except for the OGTT or test meal and a small amount of water, participants refrained from food, drinks and physical activity during the test. During the OGTT, blood samples were drawn in the fasting state and at 15, 30, 60, 90 and 120 minutes following glucose intake. The MMT was a standardized mixed breakfast consisting of 2 croissants (90g), 10g butter, 40g cheese, 150g full-fat milk and 100g of yoghurt drink enriched with 10g of maltose. The total nutrient content was 3487kJ (74g (36 Energy%) carbohydrates, 49g (52 Energy%) fat of which 28g was saturated and 24g (12 Energy%) proteins). Blood samples were collected in fasting state and at 15, 30, 60, 90, 120, 180 and 240 minutes following meal intake. Blood samples were drawn from the antecubital vein, immediately processed after sampling and stored at -80C°.

After 7 years of follow-up, a physical examination was performed, including measurement of fasting plasma glucose levels, lifestyle factors and anthropometry. For the measurement of fasting plasma glucose levels a blood sample was taken after an overnight fast.

### GIP and GLP-1 levels

The blood samples drawn during the OGTT and MMT were assessed for GIP and GLP-1 levels. Total GLP-1 levels were determined using RIA (antiserum 89390) against standards of synthetic GLP-1 7–36 amide. The assay measures the sum of intact GLP-1 and the primary metabolite, GLP-1 9–36 amide[33]. Total GIP levels were measured using the COOH terminally directed antiserum R65, which fully reacts with intact human GIP and the NH2 terminally truncated metabolite[34]. For both assays, sensitivity was below 1pmol/l and intra-assay coefficients of variation below 6% at 20 pmol/l. In order to minimize inter-assay variation, all samples were run consecutively and without delay, using identical reagents and protocols.

### Outcome

Primary outcome was defined as the change in fasting plasma glucose levels (continuous outcome) during a median 7.0±0.2 year follow-up. Fasting plasma glucose levels at baseline and follow-up were determined using the glucose hexokinase method Modular analytics, Roche diagnostics, Mannheim, Germany, with intra- and inter-assay coefficients of variation of 1.5% and 1.5%, respectively.

### Other measures

Age and sex were self-reported. We measured weight and height, with participants wearing light clothes only, and the BMI was calculated as weight/height squared (kg/m^2^). Waist circumference was measured according to a standardised procedure, as described earlier[35]. Sitting blood pressure was measured twice on the right arm with a random-zero sphygmomanometer (Hawksley–Gelman, Lancing, UK) and the average was used. Level of insulin was measured from a fasted blood sample by immunometric assays (ACS Centaur, Bayer Diagnostics, Mijdrecht, The Netherlands). The insulinogenic index was calculated by dividing the increment in insulin during the first 30 min of OGTT/MMT by the increment in glucose over the same period [36]. HOMA-IR was calculated using the following equation: HOMA-IR = (fasting plasma insulin × fasting plasma glucose) / 22.5 [37].

### Statistical analyses

#### Characteristics of study participants

First, possible selection bias was investigated by comparing age, sex, BMI, fasting plasma glucose and 2h plasma glucose levels at baseline between participants who participated (n = 126) in the follow-up examination and those who did not (n = 82), using Student’s t-test for continuous variables and Chi-Square tests for dichotomous variables.

Baseline characteristics of the final study population (n = 121) were presented. Continuous data were presented as mean ± standard deviation (SD) or median (interquartile range (IQR)) when variables were skewed, while dichotomous data were presented as %.

#### Analysis of GIP and GLP-1 levels

GIP and GLP-1 responses following OGTT and MMT were measured as the total area under the curve (tAUC) as well as the incremental area under the curve (iAUC), the latter only measuring the area above baseline level. The AUC was calculated using the trapezoid method for the total duration of the OGTT and MMT, 2 hours and 4 hours, respectively. Linearity was checked for continuous determinants. Due to the non-linear associations that were observed, AUC data were grouped into tertiles.

#### Associations with fasting plasma glucose levels at baseline

To examine the cross-sectional association between the tAUC and iAUC of the incretin hormones and fasting glucose levels at baseline, unstandardized regression coefficients were estimated, using linear regression analyses. Although age, sex and BMI were not associated with incretin responses at the baseline measurement[29], we adjusted for these variables in our analysis because BMI, age and sex are known as important determinants of glucose levels and diabetes[16, 20]. We also assessed whether sex was a possible effect modifier.

#### Associations with fasting plasma glucose levels after 7 years of follow-up

To examine the prospective association between the tAUC and iAUC of the incretin hormones and changes in fasting glucose levels during 7 years of follow-up, unstandardized regression coefficients were estimated, using linear regression analyses. While fasting glucose levels at follow-up were expected to be higher in participants with already higher fasting glucose levels at baseline, the so called ‘horse-racing effect’, we adjusted for fasting glucose levels at baseline in all models and subsequently for age, sex, follow-up duration and BMI (model 2). In additional analyses, we adjusted for possible mediating factors; insulinogenic index as a marker of beta-cell function and HOMA-IR as a marker of insulin sensitivity (model 3). Due to the limited number of participants, we restricted the number of confounders or mediators.

To assess possible selection bias due to drop-outs at follow-up, we performed a sensitivity analysis using inverse probability weighting (IPW) analysis, based on propensity scores, to adjust for baseline differences between participants who participated in the follow-up examination and those who did not. With IPW, a logistic regression analysis is performed to estimate the probability of participating at follow-up for a particular individual and then the inverse of the predicted probability is used as a weight in the subsequent linear regression analysis. Individuals who are less likely to be present at follow-up, based on their baseline characteristics, are weighted most heavily in the subsequent linear regression analysis.

Finally, we also examined the prospective association of the fasting, 30 minute and 2h incretin levels separately with changes in fasting glucose levels during 7 years of follow-up. For these additional analyses, the same models were used as for the main analyses.

#### Associations with BMI and waist after 7 years of follow-up

Along with its insulinotropic effects, GIP enhances fat deposition and GLP-1 reduces food intake and body weight, thereby potentially exacerbating and improving obesity, respectively[38, 39]. Therefore, we also examined the prospective association of incretin levels with changes in BMI and waist circumference during 7 years of follow-up. The analyses were adjusted for BMI or waist circumference at baseline and subsequently for age, sex and follow-up duration.

Statistical analyses were performed with SPSS version 20.0 (SPSS Inc., Chicago, IL) and a p-value below 0.05 was considered to be statistically significant.

## Results

### Characteristics of study participants

Compared to the 121 participants who took part in the follow-up examination after a median 7.0±0.2 year follow-up, those who declined participation had a significantly higher BMI (28.6 vs. 26.9 kg/m^2^, p = 0.01), waist circumference (99 vs. 94 cm, p = 0.006), fasting glucose levels (6.0 vs. 5.5 mmol/l, p = 0.001) and 2h plasma glucose levels (6.6 vs. 5.2 mmol/l, p = 0.001) at baseline. No significant age and sex differences were observed.

The baseline characteristics of the study population (n = 121), including levels of fasting, tAUC and iAUC concentrations of GLP-1 and GIP, are described in Table 1. Of the 121 participants without T2DM at baseline, 9 participants (7.4%) had developed T2DM after 7 years of follow-up.

**Table 1:** Population characteristics at baseline (n = 121).

Sex (% female)	50.4
Age (years)	54.1 ± 6.6
Body mass index (kg/m^2^)	26.7 ± 3.7
Waist circumference (cm)	93.1 ± 11.2
Systolic blood pressure (mm/Hg)	134.7 ± 15.8
Diastolic blood pressure (mm/Hg)	76.8 ± 9.3
Fasting glucose (mmol/l)	5.4 ± 0.4
2h glucose OGTT (mmol/l)	5.0 ± 1.3
Fasting GIP (pmol/l)	6.3 (6.3)
Fasting GLP-1 (pmol/l)	11.0 (4.8)
Fasting insulin (pmol/l)	40.9 (30.4)
tAUC GIP OGTT (mmol/l*min)	82.3 (59.9)
tAUC GLP-1 OGTT (mmol/l*min)	34.9 (19.5)
tAUC GIP MMT (mmol/l*min)	299.2 (184.1)
tAUC GLP-1 MMT (mmol/l*min)	64.4 (29.5)
iAUC GIP OGTT (mmol/l*min)	72.3 (56.3)
iAUC GLP-1 OGTT (mmol/l*min)	12.0 (12.5)
iAUC GIP MMT (mmol/l*min)	275.8 (176.6)
iAUC GLP-1 MMT (mmol/l*min)	23.9 (26.9)
Hypertension medication (n, %)	8, 7
Lipid lowering medication (n, %)	19,16

Values are mean ± SD, or median (interquartile range) in case of a skewed distribution.

Duration of OGTT is 2h, duration MMT is 4h. tAUC = total area under the curve, iAUC = incremental area under the curve.

### Associations with fasting glucose levels at baseline

Results of the linear regression analyses between the tAUC and iAUC of GIP and GLP-1 and fasting glucose levels at baseline are shown in S1 and S2 Tables. Sex was not an effect modifier. No significant associations were observed for GIP levels. For tAUC GLP-1 following MMT, but not following OGTT, we observed that in the crude model, those with GLP-1 responses in the middle and highest tertile had significantly lower fasting glucose levels compared to those in lowest tertile (-0.28 (95% CI: -0.47; -0.08) mmol/l and -0.27 (95% CI: -0.46; -0.08) mmol/l, respectively). Similar results were observed after additional adjustment for age, sex and BMI. For iAUC GLP-1, a significant association was observed, in the crude model, for the highest as compared to the lowest tertile following OGTT, but not MMT (-0.23 (95% CI: -0.42;–0.04) mmol/l). However this association was no longer significant after adjustment for additional confounders.

### Associations with changes in fasting glucose levels during 7 years of follow-up

The mean change in fasting glucose levels during 7 years of follow-up was 0.43 ± 0.5 mmol/l (p = 0.000), resulting in a mean fasting glucose level at follow-up of 5.83 ± 0.7 mmol/l. Results of the linear regression analyses between the tAUC and iAUC of GIP and GLP-1 at baseline and changes fasting glucose levels after 7 years of follow-up are shown in Tables 2 and 3. Sex was not an effect modifier. No significant associations were observed for GIP levels. However, individuals in the lowest tertile of iAUC GLP-1 following OGTT had an increase of 0.95 mmol/l in fasting glucose levels during 7 years of follow-up. Those in the middle and highest tertiles had a significantly smaller increase (in fact, decreases) in fasting glucose levels during follow-up, adjusted for fasting glucose levels at baseline, compared to those in lowest tertile; -0.28 (95% CI: -0.54;–0.03) mmol/l and -0.38 (95% CI: -0.65;–0.12) mmol/l, respectively (Table 3). Similar results were observed after adjustment for additional confounders; age, sex, follow-up duration and BMI. Further adjustment for possible mediating variables; insulinogenic index and HOMA-IR also did not affect the associations. For the tAUC GLP-1, the same trend was observed; following OGTT, those in the highest tertile had a significantly smaller increase in fasting glucose levels (Table 2). No significant associations were observed for GLP-1 levels following the MMT.

**Table 2:** Regression coefficients (with 95% confidence intervals) for the association of the tAUC of GIP and GLP-1 following OGTT and MMT at baseline and change in fasting plasma glucose levels during 7.0 years of follow-up.

	Model 1	Model 2	Model 3
**GIP tAUC OGTT**	**N = 107**	** N = 107**	**N = 105**
Low (reference)	0.51 (-0.92; 1.95)	0.48 (-3.47; 4.43)	0.93 (-3.40; 5.26)
Middle	-0.02 (-0.29; 0.25)	-0.06 (-0.33; 0.21)	-0.05 (-0.32; 0.22)
High	-0.09 (-0.35; 0.18)	-0.07 (-0.34; 0.21)	-0.02 (-0.29; 0.25)
**GIP tAUC MMT**	**N = 106**	** N = 106**	**N = 99**
Low (reference)	0.67 (-0.70; 2.03)	0.61 (-2.94; 4.16)	0.70 (-3.07; 4.48)
Middle	0.14 (-0.12; 0.40)	0.14 (-0.12; 0.40)	0.15 (-0.12; 0.42)
High	-0.03 (-0.29; 0.23)	0.01 (-.26; 0.28)	0.04 (-0.25; 0.32)
**GLP-1 tAUC OGTT**	**N = 105**	** N = 105**	**N = 103**
Low (reference)	0.64 (-0.80; 2.08)	0.53 (-3.43; 4.49)	1.12 (-3.16; 5.40)
Middle	-0.15 (-0.41; 0.11)	-0.15 (-0.41; 0.12)	-0.12 (-0.37; 0.14)
High	**-0.33 (-0.59; -0.06)**	**-0.32 (-0.61; -0.04)**	**-0.31 (-0.60; -0.03)**
**GLP-1 tAUC MMT**	** N = 107**	** N = 107**	**N = 100**
Low (reference)	1.24 (-0.21; 2.68)	0.89 (-2.56; 4.34)	1.03 (-2.64; 4.71)
Middle	-0.19 (-0.46; 0.07)	-0.22 (-0.49; 0.05)	-0.19 (-0.47; 0.09)
High	-0.21 (-0.48; 0.05)	-0.22 (-0.48; 0.04)	-0.22 (-0.49; 0.05)

Note that only for the ‘middle’ and ‘high’ categories regression coefficients are presented. For the ‘low’ category intercepts are presented. Note that individual parameters have missing data.

Models

1: Adjusted for fasting glucose levels at baseline.

2: Adjusted for fasting glucose levels at baseline, age, sex, follow-up duration and BMI.

3: Adjusted for fasting glucose levels at baseline, age, sex, follow-up duration, BMI, insulinogenic index and HOMA-IR.

Bold = significant association.

**Table 3:** Regression coefficients (with 95% confidence intervals) for the association of the iAUC of GIP and GLP-1 following OGTT and MMT at baseline and change in fasting plasma glucose levels during 7.0 years of follow-up.

	Model 1	Model 2	Model 3
**GIP iAUC OGTT**	**N = 107**	** N = 107**	**N = 105**
Low (reference)	0.54 (-0.91; 1.20)	0.61 (-3.34; 4.56)	1.06 (-3.35; 5.37)
Middle	-0.03 (-0.29; 0.24)	-0.09 (-0.37; 0.18)	-0.08 (-0.36; 0.19)
High	-0.05 (-0.32; 0.21)	-0.05 (-0.32; 0.22)	-0.003 (-0.27; 0.26)
**GIP iAUC MMT**	**N = 106**	** N = 106**	**N = 99**
Low (reference)	0.80 (-0.58; 2.19)	0.64 (-2.95; 4.22)	0.82 (-2.99; 4.64)
Middle	-0.07 (-0.34; 0.19)	-0.06 (-0.33; 0.21)	-0.01 (-0.29; 0.27)
High	-0.08 (-0.34; 0.18)	-0.02 (-0.29; 0.26)	-0.04 (-0.32; 0.25)
**GLP-1 iAUC OGTT**	**N = 105**	** N = 105**	**N = 103**
Low (reference)	1.07 (-0.41; 2.56)	0.95 (-3.00; 4.91)	1.06 (-3.23; 5.34)
Middle	**-0.28 (-0.54; -0.03)**	**-0.28 (-0.54; -0.01)**	-0.27 (-0.53; 0.001)
High	**-0.38 (-0.65; -0.12)**	**-0.39 (-0.68; -0.10)**	**-0.35 (-0.63; -0.06)**
**GLP-1 iAUC MMT**	** N = 107**	** N = 107**	**N = 100**
Low (reference)	0.92 (-0.48; 2.32)	1.11 (-2.37; 4.58)	1.14 (-2.56; 4.84)
Middle	0.11 (-0.15; 0.37)	0.14 (-0.14; 0.41)	0.07 (-0.21; 0.35)
High	-0.01 (-0.27; 0.24)	0.05 (-0.22; 0.32)	0.03 (-0.26; 0.32)

Note that only for the ‘middle’ and ‘high’ categories regression coefficients are presented. For the ‘low’ category intercepts are presented. Note that individual parameters have missing data.

Models

1: Adjusted for fasting glucose levels at baseline.

2: Adjusted for fasting glucose levels at baseline, age, sex, follow-up duration and BMI.

3: Adjusted for fasting glucose levels at baseline, age, sex, follow-up duration, BMI, insulinogenic index and HOMA-IR.

Bold = significant association.

In the sensitivity analyses using IPW, the association of the tAUC and iAUC of GLP-1 following OGTT with the change in fasting glucose levels during follow-up became more pronounced. For the iAUC of GLP-1, the regression coefficients, in the baseline glucose, age, sex, follow-up duration and BMI adjusted model, for those in the middle and highest tertiles, compared to those in lowest tertile, were; -0.30 (95% CI: -0.57; -0.03) and -0.42 (95% CI:-0.71; -0.12), respectively. For the tAUC of GLP-1, the regression coefficients were; -0.16 (95% CI: -0.42; 0.11) and -0.36 (95% CI: -0.65; -0.06), respectively. Furthermore, excluding individuals who were on lipid lowering or antihypertensive medication, did not change the estimates. For the iAUC of GLP-1, the regression coefficients, adjusted for baseline glucose, age, sex, follow-up duration and BMI, for those in the middle and highest tertiles, compared to the lowest tertile, were; -0.29 (95% CI: -0.60; -0.02) and -0.40 (95% CI: -0.74; -0.06), respectively. For the tAUC of GLP-1, the regression coefficients were; -0.18 (95% CI: -0.47; 0.12) and -0.34 (95% CI: -0.69; 0.01), respectively.

When the association between incretin response and changes in fasting glucose levels was examined, using only the fasting, 30 minute or 2h OGTT levels instead of the AUC, no significant associations were observed. These results suggested that incretin levels at single time points were not predictive and cannot be used instead of the AUC of the incretin response. The regression coefficients for the middle and highest tertile of fasting GLP-1, compared to the lowest tertile, adjusted for fasting glucose levels at baseline, were; 0.03 (95% CI: -0.20; 0.27) and -0.13 (95% CI:-0.36; 0.11), respectively. For the fasting levels of GIP, the regression coefficients were; 0.09 (95% CI: -0.14; 0.32) and -0.05 (95% CI: -0.30; 0.19), respectively. The regression coefficients for the middle and high tertile of 30 minute OGTT levels of GLP-1, compared to the lowest tertile, adjusted for fasting glucose levels at baseline, were; -0.10 (95% CI: -0.35; 0.15) and -0.20 (95% CI: -0.44; 0.04), respectively. For the 30 minute OGTT levels of GIP, the regression coefficients were; 0.02 (95% CI: -0.22; 0.27) and -0.02 (95% CI: -0.28; 0.22), respectively. Finally, the regression coefficients for the middle and highest tertile of 2h OGTT levels of GLP-1, compared to the lowest tertile, adjusted for fasting glucose levels at baseline, were; 0.16 (95% CI: -0.09; 0.41) and -0.10 (95% CI: -0.35; 0.14), respectively. For the 2h OGTT levels of GIP, the regression coefficients were; -0.05 (95% CI: -0.29; 0.20) and 0.09 (95% CI: -0.15; 0.34), respectively.

### Associations with BMI and waist during 7 years of follow-up

Results of the linear regression analyses between the tAUC and iAUC of GIP and GLP-1 at baseline and changes in BMI and waist circumference after 7 years of follow-up are shown in S3 and S4 Tables. In general, only small differences were observed between the groups. For GIP, no significant associations were observed with changes in BMI and waist circumference. For GLP-1, compared to those in the lowest tertile of the tAUC following MMT, participants in the highest tertile had a statistically significant smaller increase in BMI; -1.03 kg/m2 (95% confidence interval: -1.89;-0.16) mmol/l (S3 Table). The same was observed for the iAUC of GLP-1 following OGTT; those in the highest tertile had a statistically significant smaller increase in BMI; -1.00 kg/m2 (95% confidence interval: -1.91;-0.09). Although participants in the highest GLP-1 tertile also seemed to have a smaller increase in waist circumference, except for iAUC GLP-1 following MMT (S4 Table), no significant associations were observed between GLP-1 and changes in waist circumference.

## Discussion

To our knowledge, this study is the first prospective study to examine the association of incretin responses to an OGTT and MTT at baseline and the change in fasting plasma glucose during 7 years of follow-up, in individuals who were non-diabetic at baseline. Up until now, the cross-sectional evidence on the aetiological role of the incretin hormones in T2DM has been inconclusive[10, 13–26, 29, 40]. In our current prospective analyses (without T2DM patients) GIP response was not associated with fasting glucose levels at baseline or with a change in fasting glucose levels during follow-up. In contrast, for GLP-1 both the cross-sectional baseline as the prospective analyses showed an inverse association between GLP-1 and (change in) fasting glucose, suggesting that a low GLP-1 response was predictive for glucose deterioration.

Comparison of our results to previous studies is hampered by the cross-sectional design of those studies[10, 13–26, 29]. Up until now, the predominant view was that altered incretin secretion is unlikely to be an initial defect in the aetiology of T2DM and instead is a consequence of the diabetic state[17, 40–42]. For example, in a study of identical twins, discordant for diabetes, only the one with T2DM showed impaired GLP-1 secretion, suggesting that an impairment in GLP-1 secretion may be a consequence of T2DM[17]. However, a recent study of Færch et al. (2015) challenged this view, showing that the GLP-1 response to an OGTT was impaired in subjects with pre-diabetes, suggesting that changes in GLP-1 secretion precede glucose deterioration rather than being a consequence of it[20]. Although results of previous cross-sectional studies should be interpreted carefully, the results from Færch et al. (2015) are in line with ours, which indicate that reduced GLP-1 secretion does indeed predict glucose deterioration. While our study is the first prospective study, additional studies are necessary to replicate these results.

Our current observation that only the secretion of GLP-1 and not GIP was significantly associated with a change in fasting glucose levels during follow-up can be seen as unexpected, as the two incretin hormones are very similar with respect to insulinotropic effects, i.e. receptors and signal transduction mechanisms[43, 44]. However, GLP-1 and GIP have dissimilar metabolic effects, with GLP-1 being more favorable for glucose homeostasis and energy balance, as GLP-1 directly inhibits glucagon and increases satiety and thereby reduces energy intake[6, 38]. In contrast, GIP is involved in energy storage and fat deposition, which when chronic, promotes diabetes development[45]. In our analyses, an association was observed for GLP-1 but not for GIP with changes in BMI over time, with high levels of GLP-1 being associated with a smaller increase in BMI, compared to those with low levels of GLP-1. This is in line with previous studies showing that GLP-1 reduces food intake and body weight [38, 39]. While our study is the first prospective study, we need additional studies to confirm these results and provide possible (metabolic) explanations for the observation that only the secretion of GLP-1 and not GIP was associated with changes in fasting glucose levels.

The second unexpected result was the absence of a significant association between GLP-1 response and glucose concentrations at follow-up after a MMT, which was significant for the response after an OGTT. This discrepancy might be explained by the overall lower and slower incretin response to the MMT as compared to the OGTT, making it harder to distinguish differences. Finally, we observed differences in association for the tAUC versus the iAUC. For tAUC, the association following the MMT was similar to that of the OGTT, albeit not significant, while this was not the case for iAUC. This difference could be explained by the fact that the tAUC is predominantly dependent on the fasting levels, which are expected to be very similar between the MMT and OGTT[27]. As the iAUC is more dependent on the load and the response is thus more dissimilar between the MMT and OGTT, this possibly explains our observed difference. However, as our study is the first prospective study, additional studies are necessary to support these results.

Our study has some limitations. First, the number of subjects investigated in this study is rather limited and studies with larger sample sizes are required to confirm our results. However, we feel that the need for a population-based, prospective study, on this topic outweighs the limited number of subjects. Second, because only 9 participants developed T2DM at follow-up, we were unable to assess the association between the incretins and incident T2DM, due to lack of statistical power. Third, while we have not conducted OGTT’s or determined insulin and glucagon levels at follow-up and HbA1c data is lacking at baseline, our analyses are limited to (change in) fasting glucose levels at follow-up, not reflecting total glucose metabolism or insulin sensitivity status. Fourth, our results might also be explained by an age dependent decline of GLP-1 response. However, our analyses were corrected for age at baseline and in addition, Færch et al. (2015) showed a positive association between age and GLP-1 response, not a negative one[20]. Fifth, as with any observational prospective study, the baseline measurement of the exposure, the incretin response, may not reflect the exposure during the (entire) follow-up period, and may have changed upon factors such as diet or medication. Unfortunately no information on changes of diet or drug exposure during follow-up was available. Finally, the participants included in the analysis were significantly healthier compared to the participants who declined participation at follow-up and this could have led to an underestimation of observed associations. Indeed, in a sensitivity analysis using IPW to adjust for selection by such drop-outs, the association of the tAUC and iAUC of GLP-1 following OGTT with the change in fasting glucose levels during follow-up became more pronounced.

The main strength of this study is that we were able to conduct a prospective study on the association of the incretin hormones with change in fasting glucose levels. Second, extensive baseline measures were conducted, including an OGTT as well as a MMT, which provides additional information on the normal physiological response after eating a meal. Third, analyzing a generally healthy population at baseline reduces the role of major potential confounders such as variations in the rate of gastric emptying. In addition, adjusting for possible confounders or mediating variables, including indicators of insulin sensitivity or beta-cell function, or overweight, did not explain the observed associations. Neither did exclusion of individuals who were using antihypertensive or lipid lowering drugs at baseline. Fourth, incretin analyses were made at the time of plasma sampling. Any other study design would have caused problems because of long term storage of plasma samples (retrospective studies) or because of a 3 times greater inter-assay variation than intra-assay variation (prospective studies). Finally, we measured plasma total GIP and GLP-1 levels and not plasma intact GIP and GLP-1 levels. Plasma concentrations of total GIP and GLP-1 include the intact, active hormone and the inactive primary metabolites of GIP and GLP-1 and are therefore better indicators of the overall secretory response than plasma intact incretin levels as these are rapidly degraded by the enzyme DPP4 [12].

In conclusion, the current study suggests a low GLP-1 response to an OGTT to be associated with a steeper increase in fasting plasma glucose levels over time. These results support the hypothesis that reduced GLP-1 secretion precedes glucose deterioration and this reduction might play a role in the etiology of T2DM.

## Supporting information

S1 Table Regression coefficients (with 95% confidence intervals) for the association of the tAUC of GIP and GLP-1 following OGTT and MMT and fasting plasma glucose level at baseline. (DOCX)

S2 Table Regression coefficients (with 95% confidence intervals) for the association of the iAUC of GIP and GLP-1 following OGTT and MMT and fasting plasma glucose level at baseline. (DOCX)

S3 Table Regression coefficients (with 95% confidence intervals) for the association of the tAUC of GIP and GLP-1 following OGTT and MMT at baseline and change in BMI and waist during 7.0 years of follow-up. (DOCX)

S4 Table Regression coefficients (with 95% confidence intervals) for the association of the iAUC of GIP and GLP-1 following OGTT and MMT at baseline and change in BMI and waist during 7.0 years of follow-up. (DOCX)
