# Supplementary material for: Incretin responses to oral glucose and mixed meal tests and changes in fasting glucose levels during 7 years of follow-up: The Hoorn Meal Study
Source: PLoS One. 2018 Jan 11;13(1):e0191114. doi: 10.1371/journal.pone.0191114 (PMC5764355; doi:10.1371/journal.pone.0191114)
Supplement: S1 Table — (DOCX) [file pone.0191114.s001.docx]

**S1 Table.** Regression coefficients (with 95% confidence intervals) for the association of the tAUC of GIP and GLP-1 following OGTT and MMT and fasting plasma glucose level at baseline.

|  | **Model 1** | **Model 2** |
| --- | --- | --- |
| **GIP tAUC OGTT N=107** |  |  |
| Low (reference) |  |  |
| Middle | 0.16 (-0.03 ; 0.35) | 0.18 (-0.02 ; 0.36) |
| High | 0.13 (-0.07 ; 0.32) | 0.09 (-0.09 ; 0.27) |
|  |  |  |
| **GIP tAUC MMT N=106** |  |  |
| Low (reference) |  |  |
| Middle | -0.04 (-0.24 ; 0.17) | -0.07 (-0.25 ; 0.12) |
| High | -0.03 (-0.24 ; 0.17) | -0.02 (-0.21 ; 0.17) |
|  |  |  |
| **GLP-1 tAUC OGTT N=105** |  |  |
| Low (reference) |  |  |
| Middle | 0.03 (-0.17 ; 0.22) | -0.01 (-0.18 ; 0.18) |
| High | -0.03 (-0.23 ; 0.17) | 0.02 (-0.18 ; 0.22) |
|  |  |  |
| **GLP-1 tAUC MMT N=107** |  |  |
| Low (reference) |  |  |
| Middle | **-0.28 (-0.47 ; -0.08)** | **-0.24 (-0.41 ; -0.07)** |
| High | **-0.27 (-0.46 ; -0.08)** | **-0.22 (-0.39 ; -0.05)** |

Models:

1: Crude

2: Adjusted for age, sex and BMI

Bold = significant association
