# Supplementary material for: Incretin responses to oral glucose and mixed meal tests and changes in fasting glucose levels during 7 years of follow-up: The Hoorn Meal Study
Source: PLoS One. 2018 Jan 11;13(1):e0191114. doi: 10.1371/journal.pone.0191114 (PMC5764355; doi:10.1371/journal.pone.0191114)
Supplement: S2 Table — (DOCX) [file pone.0191114.s002.docx]

**S2 Table.** Regression coefficients (with 95% confidence intervals) for the association of the iAUC of GIP and GLP-1 following OGTT and MMT and fasting plasma glucose level at baseline.

|  | **Model 1** | **Model 2** |
| --- | --- | --- |
| **GIP iAUC OGTT N=107** |  |  |
| Low (reference) |  |  |
| Middle | -0.06 (-0.26 ; 0.13) | 0.03 (-0.16 ; 0.22) |
| High | 0.01 (-0.19 ; 0.20) | 0.01 (-0.17 ; 0.19) |
|  |  |  |
| **GIP iAUC MMT N=106** |  |  |
| Low (reference) |  |  |
| Middle | -0.08 (-0.28 ; 0.13) | 0.01 (-0.18 ; 0.19) |
| High | -0.07 (-0.27 ; 0.13) | -0.01 (-0.21; 0.18) |
|  |  |  |
| **GLP-1 iAUC OGTT N=105** |  |  |
| Low (reference) |  |  |
| Middle | -0.05 (-0.24 ; 0.13) | 0.02 (-0.16 ; 0.20) |
| High | **-0.23 (-0.42 ; -0.04)** | -0.14 (-0.33 ; 0.06) |
|  |  |  |
| **GLP-1 iAUC MMT N=107** |  |  |
| Low (reference) |  |  |
| Middle | 0.24 (0.05 ; 0.43) | 0.16 (-0.02 ; 0.35) |
| High | -0.06 (-0.25 ; 0.13) | -0.08 (-0.26 ; 0.11) |

Models:

1: Crude

2: Adjusted for age, sex and BMI

Bold = significant association
