# Supplementary material for: Incretin responses to oral glucose and mixed meal tests and changes in fasting glucose levels during 7 years of follow-up: The Hoorn Meal Study
Source: PLoS One. 2018 Jan 11;13(1):e0191114. doi: 10.1371/journal.pone.0191114 (PMC5764355; doi:10.1371/journal.pone.0191114)
Supplement: S3 Table — (DOCX) [file pone.0191114.s003.docx]

**S3 Table.** Regression coefficients (with 95% confidence intervals) for the association of the tAUC of GIP and GLP-1 following OGTT and MMT at baseline and changes in BMI and waist circumference during 7.0 years of follow-up.

|  | **Change in BMI** | **Change in waist circumference** |
| --- | --- | --- |
| **GIP tAUC OGTT N=107** |  |  |
| Low (reference) | -2.91 (-15.22 ;9.40) | 18.10 (-21.10 ; 57.31) |
| Middle | -0.18 (-1.03 ; 0.67) | -1.60 (-4.28 ; 1.09) |
| High | 0.40 (-0.45 ; 1.25) | 1.69 (-1.03 ; 4.40) |
|  |  |  |
| **GIP tAUC MMT N=106** |  |  |
| Low (reference) | 1.31 (-10.49 ; 13.10) | 18.79 (-17.00 ; 54.58) |
| Middle | -0.16 (-1.06 ; 0.74) | 1.05 (-1.67 ; 3.77) |
| High | -0.54 (-1.48 ; 0.40) | -0.07 (-2.91 ; 2.78) |
|  |  |  |
| **GLP-1 tAUC OGTT N=105** |  |  |
| Low (reference) | -4.17 (-16.50 ; 8.17) | 13.16 (-27.37 ; 53.69) |
| Middle | -0.64 (-1.47 ; 0.19) | -1.02 (-3.74 ; 1.70) |
| High | -0.70 (-1.61 ; 0.21) | -1.33 (-4.31 ; 1.65) |
|  |  |  |
| **GLP-1 tAUC MMT N=107** |  |  |
| Low (reference) | 2.81 (-8.60 ; 14.21) | 20.22 (-15.48 ; 55.92) |
| Middle | -0.11 (-1.00 ; 0.78) | -0.97 (-3.77 ; 1.83) |
| High | **-1.03 (-1.89 ; -0.16)** | -1.52 (-4.23 ; 1.20) |

Note that only for the ‘middle’ and ‘high’ categories regression coefficients are presented. For the ‘low’ category intercepts are presented.

Adjusted for age, sex, follow-up duration and variable of interest at baseline.

Bold = significant association
