# Supplementary material for: Incretin responses to oral glucose and mixed meal tests and changes in fasting glucose levels during 7 years of follow-up: The Hoorn Meal Study
Source: PLoS One. 2018 Jan 11;13(1):e0191114. doi: 10.1371/journal.pone.0191114 (PMC5764355; doi:10.1371/journal.pone.0191114)
Supplement: S4 Table — (DOCX) [file pone.0191114.s004.docx]

**S4 Table.** Regression coefficients (with 95% confidence intervals) for the association of the iAUC of GIP and GLP-1 following OGTT and MMT at baseline and changes in BMI and waist circumference during 7.0 years of follow-up.

|  | **Change in BMI** | **Change in waist circumference** |
| --- | --- | --- |
| **GIP iAUC OGTT** |  |  |
| Low (reference) | 0.91 (-10.17 ; 12.00) | 19.41 (-20.22 ; 59.04) |
| Middle | -0.44 (-1.32 ; 0.45) | -1.46 (-4.27 ; 1.35) |
| High | 0.24 (-0.62 ; 1.10) | 1.42 (-1.30 ; 4.14) |
|  |  |  |
| **GIP iAUC MMT** |  |  |
| Low (reference) | 0.89 (-10.98 ; 12.75) | 16.69 (-19.30 ; 52.68) |
| Middle | -0.13 (-1.06 ; 0.81) | 0.556 (-2.26 ; 3.38) |
| High | -0.36 (-1.33 ; 0.60) | 0.47 (-2.45 ; 3.38) |
|  |  |  |
| **GLP-1 iAUC OGTT** |  |  |
| Low (reference) | -2.90 (-15.21 ; 9.41) | 13.86 (-29.92 ; 54.63) |
| Middle | -0.39 (-1.24 ; 0.45) | -1.24 (-4.01 ; 1.53) |
| High | **-1.00 (-1.91 ; -0.09)** | -1.26 (-4.25 ; 1.72) |
|  |  |  |
| **GLP-1 iAUC MMT** |  |  |
| Low (reference) | 1.30 (-10.18 ; 12.78) | 21.14 (-13.84 ; 56.12) |
| Middle | 0.26 (-0.67 ; 1.18) | 1.42 (-1.38 ; 4.21) |
| High | -0.16 (-1.09 ; 0.77) | 1.37 (-1.45 ; 4.20) |

Note that only for the ‘middle’ and ‘high’ categories regression coefficients are presented. For the ‘low’ category intercepts are presented.

Adjusted for age, sex, follow-up duration and variable of interest at baseline.

Bold = significant association
